# Supplementary material for: Atelerix algirus, the North African Hedgehog: Suitable Wild Host for Infected Ticks and Fleas and Reservoir of Vector-Borne Pathogens in Tunisia
Source: Pathogens. 2021 Jul 29;10(8):953. doi: 10.3390/pathogens10080953 (PMC8399139; doi:10.3390/pathogens10080953)
Supplement: Supplementary file 1 [file pathogens-10-00953-s001.zip › pathogens-1277226-supplementary.pdf]

Table S1. List of primers and probes sets used on the BioMark™ system (the high-throughput real-time microfluidic PCR system) for this study.

Species	Targeted Gene	Primers and Probes	Sequence 5′-3′	Length (bp)	Reference
Borrelia burgdorferi sensu stricto	rpoB	Bo_bu rpoB_F	GCTTACTCACAAAAGGCGTCTT	83	[1]
		Bo_bu rpoB_R	GCACATCTCTTACTTCAAATCCT		
		Bo_burpoB_P	AATGCTCTTGGACCAGGAGGACTTTCA		
Borrelia garinii	rpoB	Bo_ga rpoB_F	TGGCCGAACTTACCCACAAAA	83	[1]
		Bo_ga rpoB_R	ACATCTCTTACTTCAAATCCTGC		
		Bo_ga rpoB_P	TCTATCTCTTGAAAGTCCCCCTGGTCC		
Borrelia afzelii	FlaB	Bo_af fla_F	GGAGCAAATCAAGATGAAGCAAT	116	[1]
		Bo_af fla_R	TGAGCACCCTCTTGAACAGG		
		Bo_af fla_P	TGCAGCCTGAGCAGCTTGAGCTCC		
Borrelia valaisiana	ospA	Bo_va ospA_F	ACTCACAAATGACAGATGCTGAA	135	[1]
		Bo_va ospA_R	GCTTGCTTAAAGTAACAGTACCT		
		Bo_va ospA_P	TCCGCCTACAAGATTTCCTGGAAGCTT		
Borrelia lusitaniae	rpoB	Bo_lu rpoB_F	CGAACTTACTCATAAAAGGCGTC	87	[1]
		Bo_lu rpoB_R	TGGACGTCTCTTACTTCAAATCC		
		Bo_lu rpoB_P	TTAATGCTCTCGGGCCTGGGGGACT		
Borrelia spielmanii	fla	Bo_spi fla_F	ATCTATTTTCTGGTGAGGGAGC	71	[1]
		Bo_spi fla_R	TCCTTCTTGTTGAGCACCTTC		
		Bo_spi fla_P	TTGAACAGGCGCAGTCTGAGCAGCTT		
Borrelia bissettii	rpoB	Bo_bis rpoB_F	CGAACTTACTCATAAAAGGCGTC	118	[1]
		Bo_bis rpoB_R	TGGACGTCTCTTACTTCAAATCC		
		Bo_bis rpoB_P	TTAATGCTCTCGGGCCTGGGGGACT		
Borrelia miyamotoi	glpQ	Bo_miy glpQ_F	CACGACCCAGAAATTGACACA	94	[1]
		Bo_miy glpQ_R	GTGTGAAGTCAGTGGCGTAAT		
		Bo_miy glpQ_P	TCGTCCGTTTTCTCTAGCTCGATTGGG		
Borrelia spp.	23S rRNA	Bo_bu_sl_23S_F	GAGTCTTAAAAGGGCGATTTAGT	73	[1]
		Bo_bu_sl_23S_R	CTTCAGCCTGGCCATAAATAG		
		Bo_bu_sl_23S_P	AGATGTGGTAGACCCGAAGCCGAGT		
Anaplasma marginale	msp1b	An_ma msp1_F	CAGGCTTCAAGCGTACAGTG	85	[1]
		An_ma msp1_R	GATATCTGTGCCTGGCCTTC		
		An_ma msp1_P	ATGAAAGCCTGGAGATGTTAGACCGAG		
Anaplasma platys	groEL	An_pl groEL_F	TTCTGCCGATCCTTGAAAACG	75	[1]
		An_pl groEL_R	CTTCTCCTTCTACATCCTCAG		
		An_pl groEL_P	TTGCTAGATCCGGCAGGCCTCTGC		
Anaplasma phago-cytophilum	msp2	An_ph msp2_F	GCTATGGAAGGCAGTGTTGG	77	[1]
		An_ph msp2_R	GTCTTGAAGCGCTCGTAACC		
		An_ph msp2_P	AATCTCAAGCTCAACCCTGGCACCAC		
Anaplasma ovis	Msp4	An_ov msp4_F	TCATTCGACATGCGTGAGTCA	92	[1]
		An_ov msp4_R	TTTGCTGGCGCACTCACATC		
		An_ov msp4_P	AGCAGAGAGACCTCGTATGTTAGAGGC		
Anaplasma centrale	groEL	An_cen groEL_F	AGCTGCCCTGCTATACACG	79	[1]
		An_cen groEL_R	GATGTTGATGCCCAATTGCTC		
		An_cen groEL_P	CTTGCATCTCTAGACGAGGTAAAGGGG		
Anaplasma bovis	groEL	An_bov groEL_F	GGGAGATAGTACACATCCTTG	73	[2]
		An_bov groEL_R	CTGATAGCTACAGTTAAGCCC		
		An_bov groEL_P	AGGTGCTGTTGGATGTACTGCTGGACC		
Anaplasma **spp.**	16S rRNA	Ana_spp_16S_F	CTTAGGGTTGTAAAACTCTTTCAG	160	[2]
		Ana_spp_16S_R	CTTTAACTTACCAAACCGCCTAC		
		Ana_spp_16S_P	ATGCCCTTTACGCCCAATAATTCCGAACA		
Ehrlichia **spp.**	16S rRNA	Neo_mik_16S_F	GCAACGCGAAAAACCTTACCA	98	[2]
		Neo_mik_16S_R	AGCCATGCAGCACCTGTGT		
		Neo_mik_16S_P	AAGGTCCAGCCAAACTGACTCTTCCG		

Ehrlichia ruminantium	gltA	Eh_ru_gltA_F<br>Eh_ru_gltA_R<br>Eh_ru_gltA_P	CCAGAAAACTGATGGTGAGTTAG<br>AGCCTACATCAGCTTGAATGAAG<br>AGTGTAAACTTGCTGTTGCTAAGGTAGCATG	116	[2]
Neoehrlichia mikurensis	groEL	Neo_mik_groEL_F<br>Neo_mik_groEL_R<br>Neo_mik_groEL_P	AGAGACATCATTCGCATTTTGGA<br>TTCCGGTGTACCATAAGGCTT<br>AGATGCTGTTGGATGTACTGCTGGACC	96	[1]
Rickettsia conorii	23S-5S ITS	Ri_co ITS_F<br>Ri_co ITS_R<br>Ri_co ITS_P	CTCACAAAGTTATCAGGTTAAATAG<br>CGATACTCAGCAAAATAATTCTCG<br>CTGGATATCGTGGCAGGGCTACAGTAT	118	[1]
Rickettsia slovaca	23S-5S ITS	Ri_slo ITS_F<br>Ri_slo ITS_R<br>Ri_slo ITS_P	GTATCTACTCACAAAGTTATCAGG<br>CTTAACTTTTACTACAATACTCAGC<br>TAATTTTCGCTGGATATCGTGGCAGGG	138	[1]
Rickettsia massiliae	23S-5S ITS	Ri_ma ITS<br>Ri_ma ITS<br>Ri_ma ITS	GTTATTGCATCACTAATGTTATACTG<br>GTTAATGTTGTTGCACGACTCAA<br>TAGCCCCGCCACGATATCTAGCAAAAA	128	[1]
Rickettsia prowazekii	gltA	Ri_pro_gltA_F<br>Ri_pro_gltA_R<br>Ri_pro_gltA_P	CAAGTATCGGTAAAGATGTAATCG<br>TATCCTCGATACCATAATATGCC<br>ATATAAGTAGGGTATCTGCGGAAGCCGAT	151	[2]
Rickettsia aeschlimannii	ITS	Rick_aesch_ITS_F	CTCACAAAGTTATCAGGTTAAATAG<br>CTTAACTTTTACTACGATACTTAGCA<br>TAATTTTTGCTGGATATCGTGGCGGGG	134	[3]
Rickettsia andeanae	ompB	Ri_and_ompB_F<br>Ri_and_ompB_R<br>Ri_and_ompB_P	GGCGGACAGGTAACTTTTGG<br>AAGGATCATAGTATCAGGAACTG<br>ACACATAGTTGACGTTGGTACAGACGGTAC	165	[3]
Rickettsia typhi	ompB	Ri_typ_ompB_F<br>Ri_typ_ompB_R<br>Ri_typ_ompB_P	CAGGTCATGGTATTACTGCTCA<br>GCAGCAGTAAAGTCTATTGATCC<br>ACAAGCTGCTACTA-<br>CAAAAAGTGCTCAAAATG	133	[2]
Rickettsia akari	ompB	Ri_aka_ompB_F<br>Ri_aka_ompB_R<br>Ri_aka_ompB_P	GTGCTGTTGCAGGTGGTAC<br>TAAAGTAATACCGTGTAATGCAGC<br>ATTACCAGCACCGTTACCTATATCACCGG	101	[3]
Rickettsia spp.	gltA	Rick_spp_gltA_F<br>Rick_spp_gltA_R<br>Rick_spp_gltA_P	GTCGCAAATGTTCACGGTACTT<br>TCTTCGTGCATTTCTTTCCATTG<br>TGCAATAGCAAGAACCGTAGGCTGGATG	78	[2]
Bartonella henselae	pap31	Bar_he_pap31_F<br>Bar_he_pap31_R<br>Bar_he_pap31_P	CCGCTGATCGCATTATGCCT<br>AGCGATTTCTGCATCATCTGCT<br>ATGTTGCTGGTGGTGTTTCCTATGCAC	107	[1]
Bartonella spp.	ssrA	Bart_spp_ssrA_F<br>Bart_spp_ssrA_R<br>Bart_spp_ssrA_P	CGTTATCGGGCTAAATGAGTAG<br>ACCCCGCTTAAACCTGCGA<br>TTGCAAATGACAACTATGCGGAAGCACGTC	118	[2]
Francisella tularensis and *Francisella*-like endosymbionts	tul4	Fr_tu_tul4_F<br>Fr_tu_tul4_R<br>Fr_tu_tul4_P	ACCCACAAGGAAGTGTAAGATTA<br>GTAATTGGGAAGCTTGTATCATG<br>AATGGCAGGCTCCAGAAGGTTCTAAGT	76	[1]
	fopA	Fr_tu_fopA_F<br>Fr_tu_fopA_R<br>Fr_tu_fopA_P	GGCAAATCTAGCAGGTCAAGC<br>CAACACTTGCTTGAACATTTCTAG<br>AACAGGTGCTTGGGATGTGGGTGGTG	91	
Coxiella burnettii and Coxiella-like endosymbionts	Idc	Co_bu_icd_F<br>Co_bu_icd_R<br>Co_bu_icd_P	AGGCCCGTCCGTTATTTTACG<br>CGGAAAATCACCATATTCACCTT<br>TTCAGGCGTTTTGACCGGGCTTGGC	74	[1]
	IS1111	Co_bu_IS111_F<br>Co_bu_IS111_R<br>Co_bu_IS111_P	TGGAGGAGCGAACCATTGGT<br>CATACGGTTTGACGTGCTGC<br>ATCGGACGTTTATGGGGATGGGTATCC	86	
Babesia microti	CCTeta	Bab_mi_CCTeta_F<br>Bab_mi_CCTeta_R<br>Bab_mi_CCTeta_P	ACAATGGATTTTCCCCAGCAAAA<br>GCGACATTTCGGCAACTTATATA<br>TACTCTGGTGCAATGAGCGTATGGGTA	145	[1]
Babesia ovis	18S rRNA	Ba_ov_RNA18S_F<br>Ba_ov_RNA18S_R<br>Ba_ov_RNA18S_P	TCTGTGATGCCCTTAGATGTC<br>GCTGGTTACCCGCGCCTT<br>TCGGAGCGGGGTCAACTCGATGCAT	92	[1]

Babesia bigemina	18S rRNA	Ba_big_RNA18S_F<br>Ba_big_RNA18S_R<br>Ba_big_RNA18S_P	ATTCCGTTAACGAACGAGACC<br>TTCCCCCACGCTTGAAGCA<br>CAGGAGTCCCTCTAAGAAGCAAACGAG	99	[1]
Babesia bovis	CCTeta	Ba_bo_CCTeta_F<br>Ba_bo_CCTeta_R<br>Ba_bo_CCTeta_P	GCCAAGTAGTGGTAGACTGTA<br>GCTCCGTCATTGGTTATGGTA<br>TAAAGACAACACTGGGTCCGCGTGG	100	[1]
Babesia caballi	Rap1	Ba_cab_rap1_F<br>Ba_cab_rap1_R<br>Ba_cab_rap1_P	GTTGTTCGGCTGGGGCATC<br>CAGGCGACTGACGCTGTGT<br>TCTGTCCCGATGTCAAGGGGCAGGT	94	[1]
Babesia divergens	hsp70	Bab_di_hsp70_F<br>Bab_di_hsp70_R<br>Bab_di_hsp70_P	CTCATTGGTGACGCCGCTA<br>CTCCTCCCGATAAGCCTCTT<br>AGAACCAGGAGGCCCGTAACCCAGA	83	[1]
Theileria mutans	ITS	The_mu_ITS_F<br>The_mu_ITS_R<br>The_mu_ITS_P	CCTTATTAGGGGCTACCGTG<br>GTTTCAAATTTGAAGTAACCAAGTG<br>ATCCGTGAAAAACGTGCCAAACTGGTTAC	119	[2]
Theileria velifera	18S rRNA	The_ve_18S_F<br>The_ve_18S_R<br>The_ve_18S_P	TGTGGCTTATCTGGGTTCGC<br>CCATTACTTTGGTACCTAAAACC<br>TTGCGTTCCCGGTGTTTTACTTTGAGAAAG	151	[2)
Theileria **spp.**	18S rRNA	Thei_spp_18S_F<br>Thei_spp_18S_R<br>Thei_spp_18S_P	GTCAGTTTTTACGACTCCTTCAG<br>CCAAAGAATCAAGAAAGAGCTATC<br>AATCTGTCAATCCTTCCTTTGTCTGGACC	213	[3]
Hepatozoon **spp.**	18S rRNA	Hepa_spp_18S_F<br>Hepa_spp_18S_R<br>Hepa_spp_18S_P	ATTGGCTTACCGTGGCAGTG<br>AAAGCATTTTAACTGCCTTGTATTG<br>ACGGTTAACGGGGGATTAGGGTTCGAT	175	[2]
Tick **spp.**	16S rRNA	Tick_spp_16S_F<br>Tick_spp_16S_R<br>Tick_spp_16S_P	AAATACTCTAGGGATAACAGCGT<br>TCTTCATCAAACAAGTATCCTAATC<br>CAACATCGAGGTCGCAAACCATTTTGTCTA	99	[2]
Rhipicephalus sanguineus sensu lato	ITS2	Rhi_san_ITS2_F<br>Rhi_san_ITS2_F<br>Rhi_san_ITS2_F	TTGAACGCTACGGCAAAGCG<br>CCATCACCTCGGTGCAGTC<br>ACAAGGGCCGCTCGAAAGGCGAGA	110	[2]
Ixodes ricinus	ITS2	Ix_ri_ITS2_F<br>Ix_ri_ITS2_R<br>Ix_ri_ITS2_P	CGAAACTCGATGGAGACCTG<br>ATCTCCAACGCACCGACGT<br>TTGTGGAAATCCCGTCGCACGTTGAAC	77	[1]
Escherichia coli	eae	eae-F2<br>eae-R<br>eae-P	CATTGATCAGGATTTTTCTGGTGATA<br>CTCATGCGGAAATAGCCGTTA<br>ATAGTCTCGCCAGTATTCGCCACCAATACC	102	[4]

References

1. Michelet, L.; Delannoy, S.; Devillers, E.; Umhang, G.; Aspan, A.; Juremalm, M.; Chirico, J.; van der Wal, F.J.; Sprong, H.; Boye Pihl, T.P.; et al. High-Throughput Screening of Tick-Borne Pathogens in Europe. *Front Cell Infect Microbiol* **2014**, *4*, 103, doi:10.3389/fcimb.2014.00103.
2. Gondard, M.; Delannoy, S.; Pinarello, V.; Aprelon, R.; Devillers, E.; Galon, C.; Pradel, J.; Vayssier-Taussat, M.; Albina, E.; Moutailler, S. Upscaling the Surveillance of Tick-Borne Pathogens in the French Caribbean Islands. *Pathogens* **2020**, *9*, 176, doi:10.3390/pathogens9030176.
3. Sprong, H.; Fonville, M.; Docters van Leeuwen, A.; Devillers, E.; Ibañez-Justicia, A.; Stroo, A.; Hansford, K.; Cull, B.; Medlock, J.; Heyman, P.; et al. Detection of Pathogens in Dermacentor Reticulatus in Northwestern Europe: Evaluation of a High-Throughput Array. *Heliyon* **2019**, *5*, doi:10.1016/j.heliyon.2019.e01270.
4. Nielsen, E.M.; Andersen, M.T. Detection and Characterization of Verocytotoxin-Producing Escherichia Coli by Automated 5′ Nuclease PCR Assay. *J Clin Microbiol* **2003**, *41*, 2884–2893, doi:10.1128/jcm.41.7.2884-2893.2003.
